# A portable recalibration workflow for reference-based variant calling in non-human genomes

**DOI:** 10.1371/journal.pcbi.1014603

**Published:** 2026-08-20

**Authors:** Hyeonjung Lee, Sunhee Kim, Michelle Audrelia Sunartha, Chang-Yong Lee, Young-suk Lee

**Affiliations:** 1 Department of Bio and Brain Engineering, Korea Advanced Institute of Science and Technology, Daejeon, Republic of Korea; 2 Department of Industrial and Systems Engineering, Kongju National University, Cheonan, Republic of Korea; 3 Graduate School of Engineering Biology, Korea Advanced Institute of Science and Technology, Daejeon, Republic of Korea; University of Queensland, AUSTRALIA

## Abstract

A key computational step in reference-based variant calling is distinguishing true genetic variants from sequencing errors. Advanced tools and workflows have been developed to handle this by computational modelling of technical errors from the sequencing machines. However, these recalibration workflows have largely been evaluated for human data only and its exact applicability for non-human data remains unknown. Here, we conducted a systematic evaluation of variant calling on human, rice, sheep, and chickpea data, and found that existing workflows introduce unexpected statistical bias, thus leading to suboptimal variant calls for non-human data. To address this problem, we present simple guidelines for constructing a “pseudo-”database (pseudoDB) of genetic variants as a scalable and portable solution for recalibration and variant calling. With human data, our pseudoDB-based workflow performs comparably to existing dbSNP-based GATK3 workflows and those using DeepVariant, Strelka2, and FreeBayes. We extend this to other non-human genomes, namely cattle, brown bear, swan goose, African oil palm, Komodo dragon, and stevia, altogether resulting in the identification of up to 242.0% unique genetic variants. The majority of newly identified variants are within the non-coding regions, hinting at the rich diversity of genome regulation in the non-human population. Our pseudoDB-based workflow is agnostic to reference genomes and modular for easy integration with other computational workflows for human and non-human resequencing data.

## Author summary

Identifying genetic variants in human and non-human species is important across agriculture, biotechnology, ecology, and evolution. However, sequencing machines are not perfect, and they often produce errors that look like real mutations. A key challenge is to develop computational workflows that reliably filter out these errors and find true genetic variations. Here, we report that standard workflows are largely optimized for human genomes and introduce systematic biases when applied to non-human species. To solve this, we developed a simple and portable workflow called “pseudo-database” (pseudoDB). Instead of relying on external information, this approach uses the raw sequencing data to build its own internal benchmark, allowing it to reduce technical noise without needing any prior genomic knowledge. We find that the pseudoDB workflow outperforms existing approaches across a diverse range of species, including brown bear, swan goose, and stevia. Notably, we uncovered tens of thousands of variants in overlooked regions that control how genes are regulated. This work levels the playing field for non-human research and enables the optimal use of genomic technology for biodiversity conservation and improving global food security.

## 1. Introduction

The success of large-scale resequencing of the human genome has led to the resequencing of non-human genomes including cultivated plants such as rice [1,2], chickpea [3,4], as well as animals such as sheep [5,6] and cattle [7,8]. One of the main objectives of these large-scale applications for non-human research is to uncover the genetic basis of particular traits and features that may be desirable for systematic breeding and direct genetic engineering [9–11]. As of Oct. 2025, the European Variation Archive contains more than 113 million and 111 million genetic variants of cattle and sheep, respectively, which are the two most abundant non-human resources other than for human variants. Yet, the success of these non-human resequencing efforts have been relatively limited compared to human research.

One primary reason for this is the gap in the computational tools and benchmark datasets. Computational tools are essential for interpreting large amounts of genome sequencing data and distinguishing genetic variation from sequencing errors [12,13]. One example is in the identification of single-nucleotide variants. Single-nucleotide polymorphism (SNP) refers to the substitution of a single nucleotide that is present in at least 1% of the population [14] and is one of the most common types of genetic variations detected from resequencing data [15]. Identifying these single-nucleotide variants from sequencing errors is accomplished by analyzing the quality of each base call made by the sequencing machine. Computational tools such as those compiled in the Genome Analysis ToolKit (GATK) [16,17] model the machine-based quality scores to account for the technical errors such as from late sequence cycles, di-nucleotide contents, and possible manufacturing flaws [18,19].

While these tools have contributed significantly to the success of human resequencing efforts, they have not been extensively evaluated for non-human data [20–22]. For example, GATK3 was developed and benchmarked on human data only [23] and the exact applicability for non-human data is unknown. Other variant callers including FreeBayes [24], Strelka2 [25], and DeepVariant [26] have been applied to non-human data [27–29], but subsequent benchmark studies on non-human data have raised concerns of large number of false positives and false negatives [28,30]. This raises the question of whether existing approaches which have been optimized for human data are generalizable and applicable to non-human data.

One key strategy to account for systematic errors made by sequencing machines is by utilizing known databases of genetic variants. Base Quality Score Recalibration (BQSR) is a data pre-processing step in GATK3’s workflow that adjusts machine-reported base quality scores by estimating the sequencing error rate of the machine, also known as the empirical error rate [23]. Specifically, BQSR relies on an external database of known variants such as dbSNP [31] and flags single-nucleotide mismatches as possible genetic variants instead of sequencing errors. Indeed, the usage of computational recalibration and GATK3’s workflow is a standard procedure for human data [32,33], though there has been increasing reports of using other variant callers without computational recalibration [34,35]. In contrast, the majority of non-human research utilized other variant callers such as FreeBayes and DeepVariant and did not adjust the machine-reported base quality scores [29,36,37].

In this work, we examined the exact impact of the variant databases in computational recalibration and variant calling across 10 different species: human (1KGP; 1000 Genomes Project), sheep (ISGC; International Sheep Genomics Consortium), rice (3KRGP; 3000 Rice Genome Project), chickpea (ICRISAT; The International Crops Research Institute for the Semi-Arid Tropics), cattle (1KBGP; The 1000 Bull Genomes Project), brown bear (NCBI:PRJNA1139383), swan goose (NCBI:PRJNA722049), African oil palm (ENA:PRJEB21246), Komodo dragon (NCBI:PRJNA738464), and stevia (NCBI:PRJNA684944). Unexpectedly, the recalibrated base quality scores were substantially biased in non-human species, indicating that GATK3’s standard procedure for recalibration and variant calling is not applicable to non-human data. To address this limitation, we developed a database-generation method called pseudoDB that gathers potential candidates directly from resequencing data. This approach circumvents the reliance on dbSNP and other manually curated databases for recalibration and variant calling. We investigate whether the pseudoDB workflow is comparable to using dbSNP-based recalibration for human data and also software-wise portable to non-human data. This includes addressing the impact of recalibration across multiple variant callers, namely DeepVariant, FreeBayes, and Strelka2, and the possibility of overfitting when utilizing the input resequencing data for pseudoDB construction.

## 2. Results

### 2.1. Technical bias of base quality score estimation in non-human data

**Fig 1:**
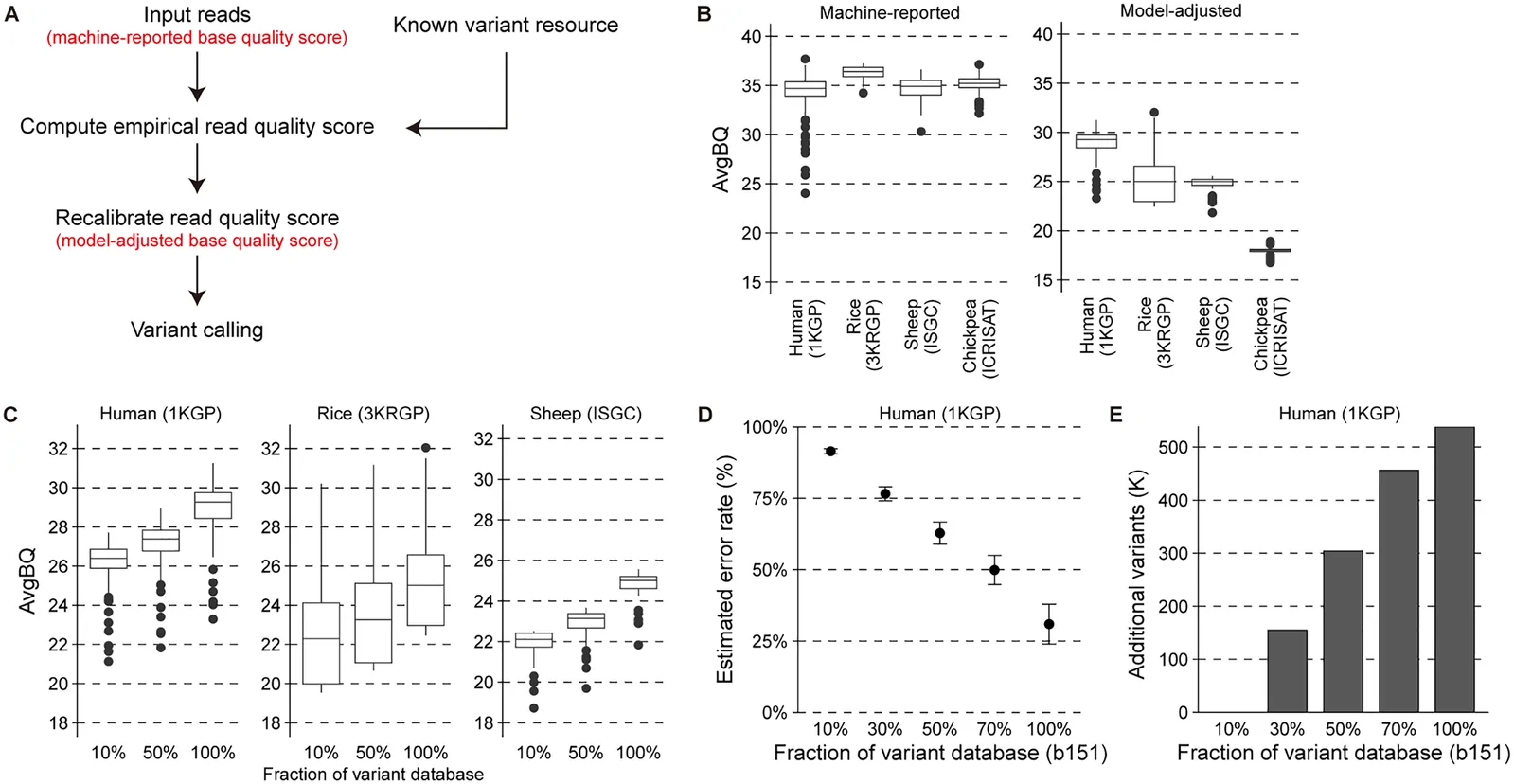
Computational recalibration of base quality scores introduces unwanted biases in model-adjusted average base quality scores (AvgBQ) and is dependent on the database version and size. (A) Computational workflow of adjusting machine-reported base quality scores for effective variant calling from genome resequencing data. (B) Distribution of AvgBQ of machine-reported and model-adjusted data across human (1KGP, n = 150), rice (3KRGP, n = 180), sheep (ISGC, n = 35), and chickpea (ICRISAT, n = 150) resequencing data. (C) fractions of the dbSNP b151 database. (D) Total empirical error rate with different fractions of the human b151 database. The points represent the mean of total empirical error rate, and the error bars indicate its standard deviation. (E) Number of additional variants called (in thousands, K) after computational recalibration using different fractions of the human b151 database is shown in a barplot.

To investigate the efficacy of base quality score recalibration (BQSR) in non-human data, we applied BQSR on published genome resequencing data from human (1000 Genomes Project, 1KGP; n = 150) [38,39], rice (Japonica; 3000 Rice Genome Project, 3KRGP; n = 180) [1], sheep (International Sheep Genomics Consortium, ISGC; n = 35) [40], and chickpea (The International Crops Research Institute for the Semi-Arid Tropics, ICRISAT; n = 150) [3] (S1 Table). Specifically, we utilized the model-adjusted quality score after recalibration as a direct measurement of its efficacy (See Method Section). Recall that BQSR is a computational method designed to account for various non-random technical errors in sequencing machines and enables sample-to-sample comparison of base quality scores [23]. Any base-level adjustments made by BQSR are considered to solely depend on the technical quality of the sequencing run (Fig 1A).

Unexpectedly, we found that the adjusted average base quality scores (AvgBQ) are dependent on the species of interest (Fig 1B). Before applying BQSR, the AvgBQ’s were fairly comparable across species (human = 34.25; rice = 36.33; sheep = 34.59; chickpea = 35.11), suggesting marginal differences in sequencing quality between human and non-human data. Of note, the outliers in the human dataset reflect the use of older sequencing platforms (e.g., Illumina Genome Analyzer II) [38]. After recalibration, the mean of AvgBQ decreased across all species (human = 28.98) but to a larger extent in rice and sheep (rice = 25.15; sheep = 24.67) and most substantially in chickpea data (chickpea = 17.98). This empirical analysis suggests that BQSR may overestimate the non-random technical errors in the non-human data compared to human data.

Of the many factors that may be responsible for this estimation bias, we hypothesized that the empirical estimation of sequencing error, also known as empirical quality score [23], may introduce statistical bias in base-level quality scores after recalibration (S1A Fig). That is, to estimate the technical error of each sample, it is crucial to determine whether a mismatch is a sequencing error or a genetic variant in the population. BQSR handles this problem by incorporating known variant databases and resources such as dbSNP [23]. The variant database acts as a whitelist for genetic variants that may have originated from the population, and BQSR uses this database to avoid overestimation of the sequencing error rate.

To measure the exact impact of the variant database, we first applied BQSR on the same set of sequencing data but using different dbSNP builds (e.g., b149, b150, and b151) (S1B Fig). Notably, chickpea was excluded from this analysis because its dbSNP records have not been updated since their initial inclusion in dbSNP build 146 (S1C-S1F Fig). Across human, rice, and sheep data, we found that the adjusted AvgBQ varies depending on the specific dbSNP build and tends to increase with more recent version updates. Subsampling the variant database by 10% and 50% resulted in a substantial decrease in AvgBQ (Fig 1C). This indicates that the size of the variant database determines the extent of overcorrection and unwanted base-level recalibration.

With only 10% of known variants, the estimated error rate was on average 91.48% (Fig 1D), which is roughly equivalent to reporting that 9 out of 10 mismatches are sequencing errors. On the contrary, we found that the estimated error rate decreases with larger databases and is 30.94% on average when utilizing the whole variant database b151. The three-fold decrease in error rate shows its strong correlation with the model-adjusted AvgBQ and results in a greater number of variants called from the sequencing data (Fig 1E). This empirical analysis suggests that existing computational pipelines using GATK3’s BQSR and UnifiedGenotyper are statistically underpowered with poor variant databases such as for non-human data. In all, these results highlight the importance of the available variant databases in computational data recalibration and suggest that re-analysis of published data with an improved variant database may lead to the discovery of new genetic variants.

### 2.2. Pseudo-database construction with an independent dataset

Unlike human data, the growth of well-known databases such as dbSNP has been relatively slow for non-human data [31]. The maintenance of these manually-curated variant databases are neither scalable nor easily adaptable for other strains or closely related species. In 2017, NCBI announced that they will no longer support non-human data in dbSNP. This limits the efficacy of existing methods for recalibration from non-human data and results in suboptimal results in variant calling.

**Fig 2:**
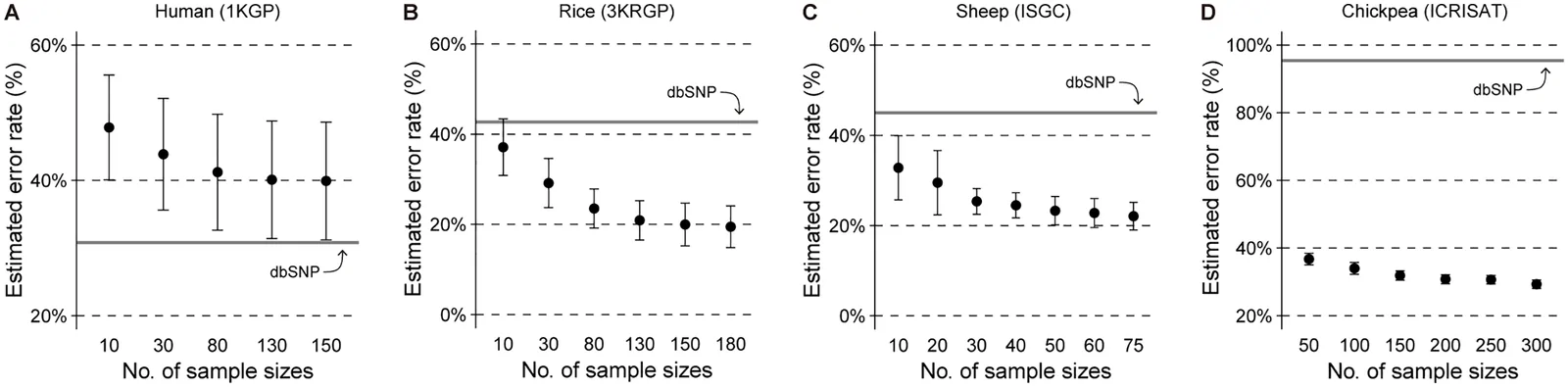
The pseudo-database (pseudoDB) construction and optimization for computational recalibration of human, rice, sheep, and chickpea resequencing data. The total empirical error rate based on pseudoDB’s that were constructed from different numbers of resequencing samples: (A) 1KGP human data, (B) 3KRGP rice data, (C) ISGC sheep data, and (D) ICRISAT chickpea data. The point represents the mean of total empirical error rate, and the error bars indicate its standard deviation. The horizontal bar represents the median total empirical error rate based on their corresponding dbSNPs.

To address this, we asked whether this suboptimal result itself may be used instead as a “pseudo”-database (pseudoDB) for base quality recalibration and variant calling, and how it should be constructed if so (S2A Fig). Specifically, an independent set of resequencing data was employed to exclude the possibility of overfitting and overestimating its performance. For example, we acquired a total of 300 human resequencing data from the 1K Genome Project (1KGP) and randomly split the data into two equal-size, disjoint sets. One key element is the number of resequencing samples used in the pseudoDB construction. With human 1KGP data, we found that the error rate decreases as more samples are used in human pseudoDB construction and converges after exceeding 100 samples (Fig 2A). This is consistent with the increase in adjusted quality scores and comparable to those obtained when using dbSNP for recalibration.

In terms of variant quality, pseudoDBs based on 80 human samples yielded 31,476 additional variants with high genotype quality (GQ ≥ 30) compared to dbSNP (S2B Fig), indicating that pseudoDB-based recalibration does not result in a decrease in high quality variants. Another factor in pseudoDB construction is an iterative approach that uses the previous pseudoDB to construct the next pseudoDB and so forth until convergence. While theoretically promising, we observed marginal improvements in the adjusted quality score with convergence occurring after the first iteration (S2C Fig), suggesting no gain in multiple rounds of pseudoDB construction.

Consistent with our analysis with human data, we also found that both the adjusted AvgBQ and error rate improves with pseudoDBs generated from an independent set of Japonica rice (*Oryza sativa*) 3KRGP data (Figs 2B and S2D), sheep (*Ovis aries*) ISGC data (Figs 2C and S2E), and chickpea (*Cicer arietinum*) ICRISAT data (Figs 2D and S2F). For Japonica rice, a pseudoDB constructed from 10 samples led to estimated error rates comparable to those obtained using dbSNP. Subsequent reductions were found up to 130 samples, suggesting that 100 samples may be as a practical rule of thumb of constructing pseudoDBs. That being said, the gain in improvement diminished beyond 50 samples for sheep and 200 samples for chickpea, indicating that systematic sample-size optimization is recommended to achieve optimal pseudoDB construction for non-human genomes. Of note, as observed with human data, no improvement was found through iterative rounds of pseudoDB construction for rice, sheep, and chickpea. Thus, we do not recommend iterative rounds for constructing pseudoDBs.

### 2.3. Sequencing depth does not resolve technical bias in human and non-human data

Recent resequencing efforts for human and non-human have demonstrated the impact of ultra-deep sequencing for variant identification, especially in terms of low-abundant variants and low-frequency mutations [21,41,42]. However, many of these studies did not apply computational recalibration nor did they report the amount of technical bias in their human or non-human data [1,3,29,38,43]. One reason for this choice might be that the amount of technical bias is marginal in ultra-deep sequencing data. That is, because higher sequencing depth is considered to be beneficial and improve the accuracy of genetic variant discovery.

To investigate the effect of sequencing depth and data quality in terms of technical bias for variant calling, we first employed the HG001 (NA12878, CEPH/Utah pedigree) pilot data from the Genome in a Bottle consortium (GIAB) [44] and compared its reported and empirical quality scores. Recall that reported quality scores refer to the machine-reported quality scores for each read group, and that the empirical quality scores refer to the sequencing accuracy based on known variant resources for each read group. For example, a root mean square error (RMSE) of 0 indicates no technical bias in sequencing quality. The HG001 data is of approximately 300x genome coverage generated by Illumina HiSeq 2500 with a machine-reported AvgBQ of >Q35. We found that RMSE between the reported and empirical quality scores of HG001 data was 3.66 (S3A Fig). Subsampling the HG001 data to 30x genome coverage resulted in a marginal increase in RMSE of 3.83, suggesting that lower genome coverage is not a major factor of technical bias in sequencing data. On the other hand, computational recalibration using dbSNP or pseudoDB led to a 3.58- to 10.35-fold reduction in RMSE in both 300x and 30x data. This indicates that both dbSNP- and pseudoDB-based approaches account for technical bias in HG001’s data regardless of sequencing depth.

**Fig 3:**
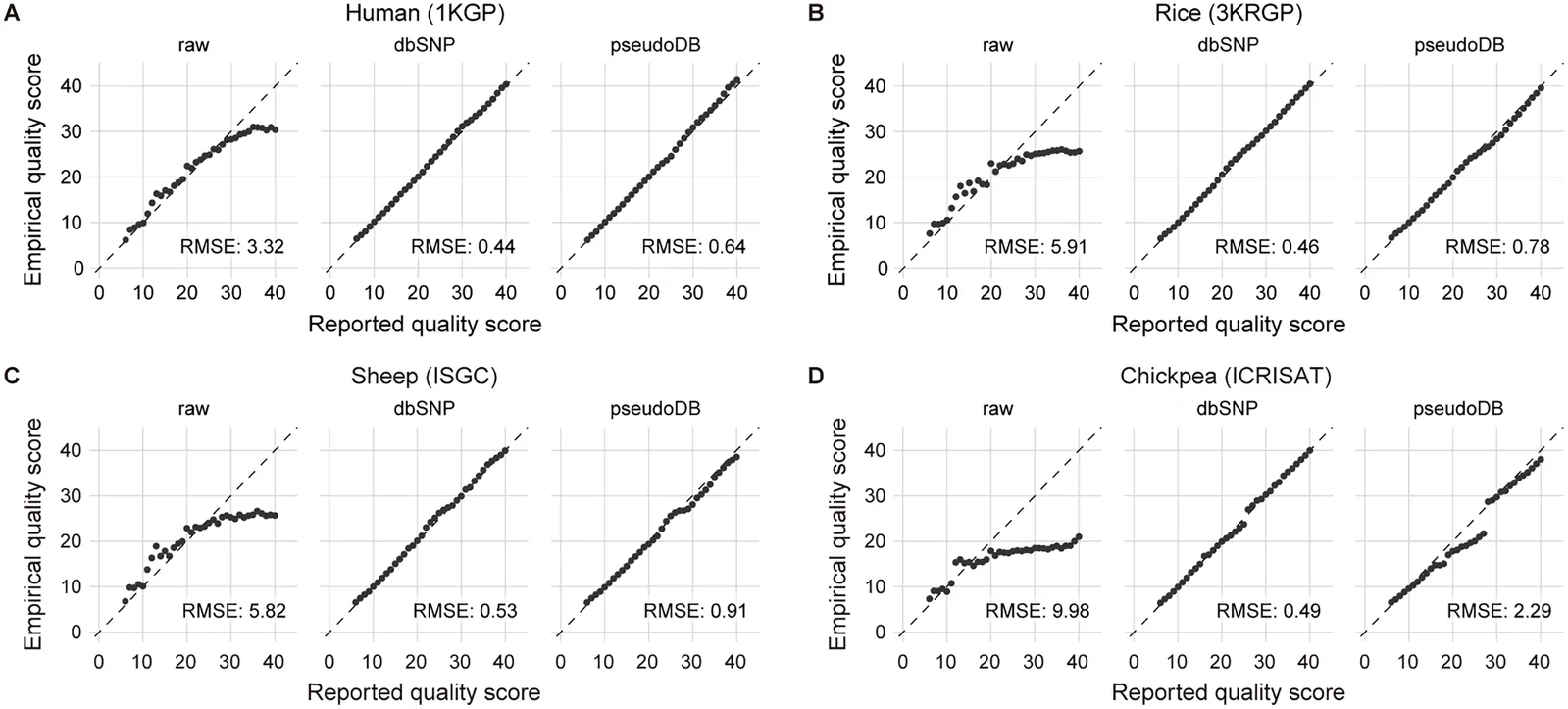
Technical noise in human and non-human resequencing data. Comparison of reported and empirical quality scores of (A) 1KGP human data (n = 150), (B) 3KRGP rice data (n = 180), (C) ISGC sheep data (n = 35), and (D) ICRISAT chickpea data (n = 150). Dashed diagonal lines indicate the identity function. RMSE: Root mean square error.

To test whether this effect generalizes to a larger cohort of human data, we measured the technical bias in 150 5x human resequencing data from 1KGP (AvgBQ > Q34). The RMSE between the reported and empirical quality scores was 3.32 for 1KGP (Fig 3A) and is comparable with the 300x and 30x GIAB data. Similar with the GIAB data analyses, computational recalibration using dbSNP and pseudoDB resulted in a 7.55- and 5.19-fold reduction in technical bias, respectively. We also constructed pseudoDBs using variant callers other than GATK3’s UnifiedGenotyper, namely DeepVariant [26], FreeBayes [24], and Strelka2 [25], and found similar reduction in technical bias in the human 1KGP data (S3B Fig). Altogether, this suggests that improved sequencing depth does not ensure low technical bias in the sequencing data and highlights the importance of computational recalibration in human data.

To evaluate the applicability of pseudoDBs to non-human genomes, we constructed pseudoDBs for Japonica rice, sheep, and chickpea and compared their performance to dbSNP-based recalibration. Specifically, we measured the technical bias in 9x Japonica rice data from 3KRGP (n = 180, AvgBQ > Q36), 13x sheep data from ISGC (n = 35, AvgBQ > Q34), and 12x chickpea data from ICRISAT (n = 150, AvgBQ > Q35). Without recalibration, RMSE were 5.91 for 3KRGP (Fig 3B), 5.82 for ISGC (Fig 3C), and 9.98 for ICRISAT (Fig 3D), highlighting the substantial technical bias irrespective of resequencing depth. dbSNP-based recalibration reduced technical bias by 12.85-fold in the 3KRGP rice data, 10.98-fold in the ISGC sheep data, and 20.37-fold reduction in the ICRISAT chickpea data. The use of pseudoDBs achieved comparable reduction, demonstrating its effectiveness in adjusting technical noise in non-human data.

In terms of the downstream analyses, the pseudoDBs yielded improved AvgBQ and lower estimated error rates (S4A-S4B Fig). Of note, the marginal improvement in chickpea AvgBQ with dbSNP-based recalibration likely reflects the limited quantity of the chickpea dbSNP database, as evidenced by its persistently high estimated error rate. More importantly, pseudoDB-based recalibration led to the discovery of additional genetic variants in Japonica rice, sheep, and chickpea (S4C Fig). In chickpea, 2,302,282 additional variants were identified with our pseudoDB workflow, which is a 126% increase compared to the standard dbSNP-based approach. Altogether, these results demonstrate that pseudoDB-based recalibration also handles the technical bias in non-human data and enhances the discovery of additional non-human variants irrespective of sequencing depth.

### 2.4. Performance of recalibration and variant calling using DeepVariant, FreeBayes, and Strelka2

The computational step of recalibration has largely been considered exclusive to GATK3’s UnifiedGenotyper [16] and unnecessary for more recent methods for variant calling including DeepVariant [26], FreeBayes [24], and Strelka2 [25]. Each variant caller improves the performance of variant identification by incorporating different modeling assumptions to handle technical bias of the resequencing data. However, the exact impact of recalibration on these variant callers has not been investigated in a quantitative and comprehensive manner.

**Fig 4:**
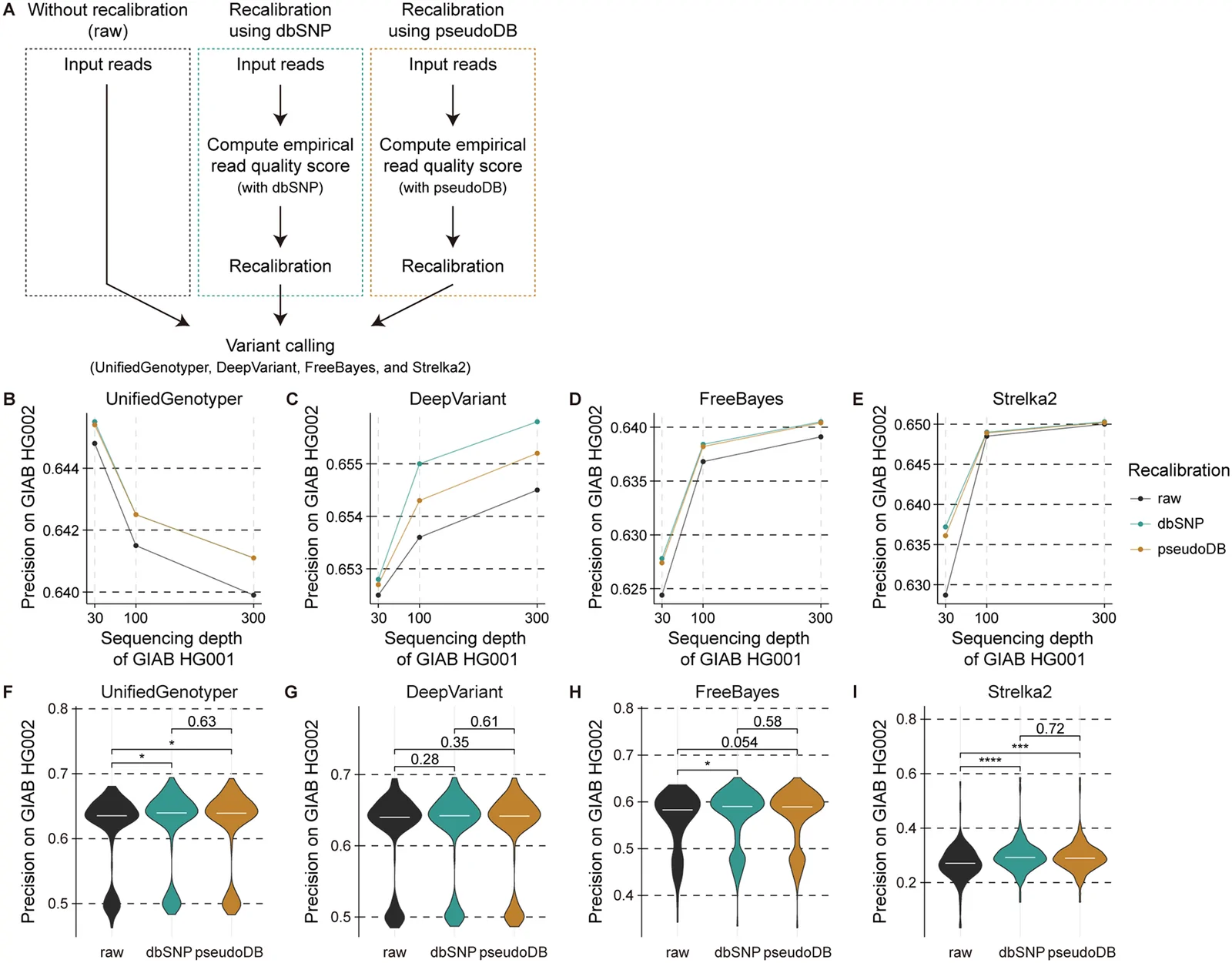
Benchmark performance of computational pipelines for recalibration and variant calling. (A) Detailed workflow for recalibration (raw in black, dbSNP-based in cyan, and pseudoDB-based in yellow) and variant calling with GATK3’s UnifiedGenotyper, DeepVariant, FreeBayes, and Strelka2. (B-E) Precision on GIAB HG002 v4.2.1 benchmark with recalibration and variant calling applied to different sequencing-depth (30x, 100x, and 300x) of GIAB HG001 human data. (F-I) Distribution of precision when applied to 1KGP human data (n = 150). White horizontal bars indicate its median precision. *P  <  0.05, **P  <  0.01, ***P  <  0.001, ****P  <  0.0001, n.s.: not significant; one-sided Wilcoxon-Mann-Whitney test.

To assess the effect of recalibration on variant callers other than GATK3’s UnifiedGenotyper, we employed human resequencing samples from the Genome in a Bottle (GIAB) project and measured the performance of variant calling in respect to their benchmark variant sets (Fig 4A). Specifically, we applied recalibration and variant calling to GIAB's HG001 resequencing data and compared the precision using GIAB's HG002 variant set as the benchmark. Recall that this human pseudoDB was constructed from the 1KGP dataset. Consistent with our previous results, precision of GATK3’s UnifiedGenotyper improved after dbSNP- and pseudoDB-based recalibration (Fig 4B). In contrast to common belief, we found improvements with recalibration for more recent variant callers including DeepVariant (Fig 4C) [26], FreeBayes (Fig 4D) [24], and Strelka2 (Fig 4E) [25]. A precision increase of <0.005 in a whole-genome analysis with millions of variants corresponds to the elimination of ten thousands of false-positive calls. This improvement was independent of resequencing depth, further highlighting the importance of recalibration for accurate variant identification.

To investigate whether this improvement is statistically significant, we applied recalibration and variant calling on an independent set of human 1KGP data (n = 150). Both dbSNP and pseudoDB-based recalibration resulted in significant improvement in the performance of GATK3’s UnifiedGenotyper compared to when used without recalibration (Fig 4F). Likewise, the precision of other variant callers significantly increased after computational recalibration (Fig 4G-4I). It is worth emphasizing that computational recalibration and greater sequencing depth may lead to a reduction in recall depending on the variant caller (S5A-S5H Fig). To avoid overestimating performance, we benchmarked using an independent HG002 variant set, as these variant sets were generated by GATK3’s UnifiedGenotyper [45] and have also been used to train other variant-calling models [25,26]. That being said, we do find a similar trend when evaluating against the HG002 variant set (S6A-S6D Fig) and likewise when benchmarking on the HG001 variant set (S6E-S6L Fig), further supporting the utility of computational recalibration independent of the variant caller.

### 2.5. Pseudo-database construction without an independent dataset and computational portability for recalibration and variant calling

**Fig 5:**
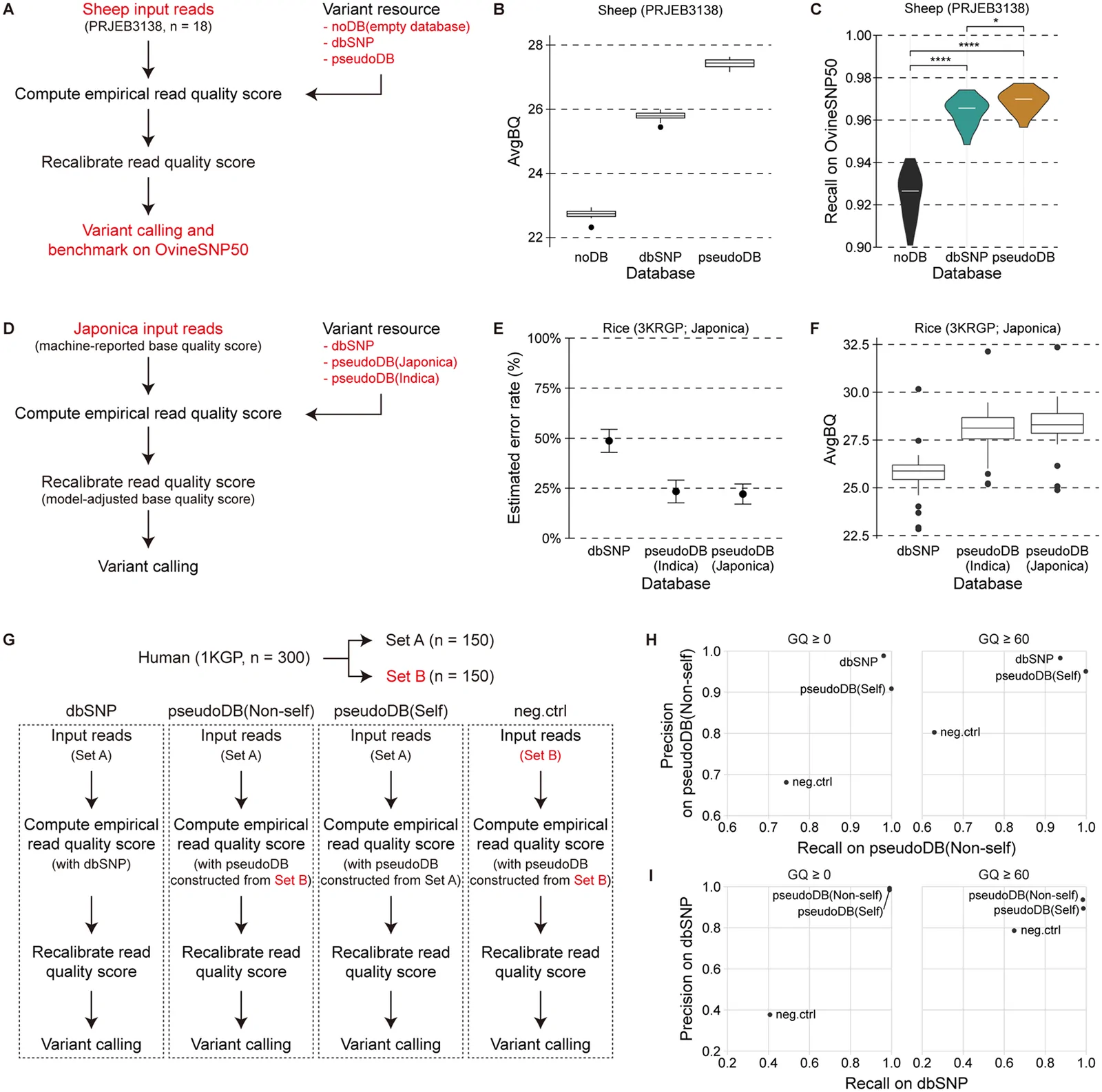
The software portability of pseudo-based approach for human and non-human data. (A) Detailed workflow for variant calling accuracy with matched OvineSNP50 and sheep resequencing data (PRJEB3138, n = 18, 13.1x genome coverage) from the Ensembl NextGen project. noDB denotes recalibration performed with an empty variant database. (B) Distribution of the adjusted AvgBQ with noDB-, dbSNP-, or pseudoDB-based recalibration. (C) Performance of variant calling with matched OvineSNP50. Distribution of recall when applied to sheep resequencing data and benchmarked on SNP array-based variants. White horizontal bars indicate its median recall. *P  <  0.05, **P  <  0.01, ***P  <  0.001, ****P  <  0.0001, n.s.: not significant; one-sided Wilcoxon-Mann-Whitney test. (D) Detailed workflow for assessing the impact of sequencing data of proximal strains used for pseudoDB construction. Japonica resequencing data (3KRGP, n = 30) was used as input for variant calling. Three variant databases were used for evaluation: dbSNP, pseudoDB constructed with an independent Japonica dataset (3KRGP, n = 130) denoted as pseudoDB(Indica), and pseudoDB constructed with Indica data denoted as pseudoDB(Indica). (E) The total empirical error rate based on dbSNP, pseudoDB(Indica), and pseudoDB(Japonica). The point represents the mean of total empirical error rate, and the error bars indicate its standard deviation. (F) Distribution of AvgBQ based on dbSNP, pseudoDB(Indica), and pseudoDB(Japonica). (G) Schematic of the data swapping analysis to quantify the effect of double-usage of input data for pseudoDB construction. 1KGP human data (n = 300) was splitted into two independent sets: Set A and B. dbSNP: Standard recalibration of Set A using the external dbSNP database. pseudoDB(Non-self): Recalibration of Set A using a pseudoDB database constructed from an independent dataset Set B. pseudoDB(Self): Recalibration of Set A using a pseudoDB database constructed from Set A itself. neg.ctrl: Recalibration of the independent dataset Set B using a pseudoDB constructed from the standard pseudoDB approach which is Set B. (H) The precision and recall of genetic variants called by the dbSNP, pseudoDB(Self), and neg.ctrl pipelines. The pseudoDB-based variants were used as the gold standard. GQ: Genotype Quality. (I) The precision and recall of genetic variants of different approaches when the dbSNP-based variants were used as the gold standard.

To directly evaluate the performance of variant calling in non-human species, we utilized matched OvineSNP50 and sheep resequencing data (PRJEB3138, n = 18, 13.1x genome coverage) from the Ensembl NextGen project (Fig 5A and S2 Table). pseudoDB-based recalibration resulted in higher AvgBQ (median of noDB = 22.7; dbSNP = 25.8; pseudoDB = 27.4) (Fig 5B), 2-fold reduction in the total empirical error rate (median of dbSNP = 0.40; pseudoDB = 0.20) (S7A Fig), and 5-fold decrease in RMSE between the reported and empirical quality scores (RMSE of raw = 5.75; pseudoDB = 1.02) (S7B Fig). Consistently, pseudoDB-based variant calling showed significant gains in recall when benchmarked on matched OvineSNP50 data (Fig 5C). Taken together, these results demonstrate that our pseudoDB-based workflow improves the accuracy of variant calling in non-human genomes, in line with the observed improvements in recalibration.

In software engineering, portability refers to the ability to be used across different computational environments and so not dependent on a single platform or resource. In terms of portability, our pseudoDB approach circumvents the computational dependency on known variant databases and offers a more portable solution for effective recalibration and subsequent variant calling. We have accomplished this by incorporating an independent set of resequencing data, and have done so to avoid the possibility of overfitting or introducing bias in pseudoDB construction. However, this independent dataset may not always be available for non-human data, limiting the portability of our pseudoDB approach.

To address this, we asked whether resequencing data of proximal strains or species may be used for pseudoDB construction. Specifically, we constructed a pseudoDB with Indica rice resequencing data from the 3000 Rice Genomes Project [1] referred to as pseudoDB(Indica) and examined its ability to recalibrate Japonica rice data (Fig 5D). Of note, dbSNP consists of known variants for Japonica rice which is considered a genome proxy for Indica rice. The pseudoDB constructed from an independent Japonica rice data referred to as pseudoDB(Japonica) was used as a positive control for this analysis. We found that the estimated error rate with pseudoDB(Indica) is comparable to pseudoDB(Japonica) (Fig 5E), indicating that Indica pseudoDB helps distinguish sequencing error from possible genetic variation in the Japonica population. The AvgBQs were also comparable after recalibration (Fig 5F), suggesting that pseudoDBs constructed from proximal data may serve as an alternative solution for handling technical bias in resequencing data for non-human data.

Next, we explored the potential of using the input data of interest for pseudoDB construction referred to as pseudoDB(Self). This double-use of data is also known as double-dipping in data analysis and not recommended in general as it opens the possibility of overfitting and overestimating the performance of the computational approach. However, the exact extent of this in the context of recalibration and variant calling remains unknown. In fact, we hypothesized that the gain might outweigh the loss in utilizing the input data for pseudoDB construction.

To measure this, we conducted a statistical data swapping analysis by first dividing 300 human 1KGP data into two equal-size, disjoint sets referred to as Set A and Set B (Fig 5G). Each set was used either as input for recalibration and/or for constructing the pseudoDB. Set A was considered the primary dataset of interest, and its variants were treated as ground truth. Set B was used as an independent dataset to construct a pseudoDB. Set A’s variants called from this workflow are referred to as pseudoDB(Non-self). pseudoDB(Self) denotes the Set A’s variants called using a pseudoDB constructed from the same Set A dataset which represents the scenario of double-use of data. As a negative control for data swapping analysis, we applied recalibration and variant calling on Set B using the pseudoDB constructed from the same dataset. Since we evaluate Set B’s variant calls against Set A’s variants as ground truth, this serves as a baseline for the data swapping analysis to establish the expected divergence when no sample-specific overlap exists.

Regarding performance, we compared the precision and recall on pseudoDB(Non-self) when applying recalibration and variant calling of variants called by using dbSNP and pseudoDB(Self) (Fig 5H). The performance of pseudoDB(Self) was comparable albeit lower across genotype quality (GQ) thresholds. The analysis of precision and recall on dbSNP-based variants revealed nearly identical performance between pseudoDB(Non-self)- and pseudoDB(Self)-based variants (Fig 5I). Additional data swapping analysis with GIAB HG001 and HG002 resequencing data (S7C Fig) revealed similar results in performance (S7D Fig). The near-identical performance between ‘Self’ (data reuse) and ‘Non-self’ (independent) directly addresses the concern regarding ‘cheating’ or genotype memorization. If the pseudoDB were simply ‘memorizing’ known variants to artificially inflate scores, the ‘Self’ configuration would show a significant performance boost over the ‘Non-self’ configuration. Instead, the parity between these two groups demonstrates that the pseudoDB workflow captures generalized technical noise and systematic error profiles rather than sample-specific genotypes. Therefore, we recommend the use of the same input when an independent dataset is not available.

### 2.6. Functional variant identification with pseudoDBs in human and non-human genomes

To further evaluate the applicability of the pseudoDB workflow to other non-human genomes including those without dbSNPs and under-studied, we apply it to 6 additional non-human species, namely cattle (*Bos taurus*; The 1000 Bull Genomes Project, 1KBGP; n = 246) [7, 46], brown bear (*Ursus arctos*; NCBI:PRJNA1139383; n = 40) [47], swan goose (*Anser cygnoides*; NCBI:PRJNA722049; n = 27) [48], African oil palm (*Elaeis guineensis*; ENA:PRJEB21246; n = 24) [49], Komodo dragon (*Varanus komodoensis*; NCBI:PRJNA738464; n = 24) [50], and stevia (*Stevia rebaudiana*; NCBI:PRJNA684944; n = 2) [51]. Along with rice, sheep, and chickpea, cattle is a high-interest species in animal breeding [52, 53]. Among the six non-human species, only cattle and African oil palm have dbSNP resources available (S8A-S8B Fig).

**Fig 6:**
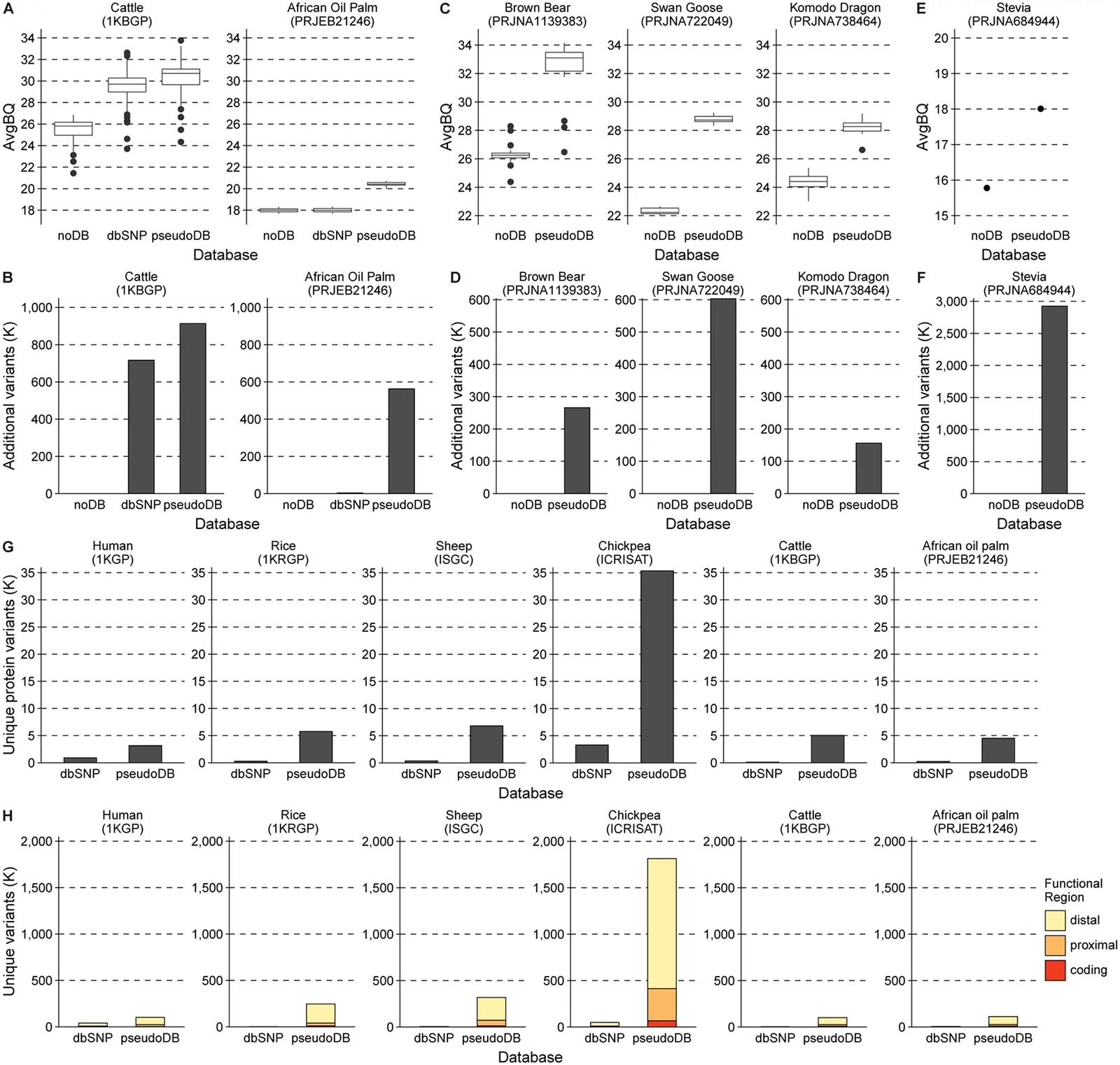
General applicability of pseudoDB workflow on other non-human species, including cattle (1KBGP, n = 146), African oil palm (PRJEB21246, n = 12), brown bear (PRJNA1139383, n = 20), swan goose (PRJNA722049, n = 14), Komodo dragon (PRJNA738464, n = 12), and stevia (PRJNA684944). (A) Distribution of the adjusted AvgBQ’s and (B) number of additional genetic variants (in thousands, K) for cattle and African oil palm. (C-D) Distribution of the adjusted AvgBQ’s and (E-F) number of additional genetic variants (in thousands, K) for brown bear, swan goose, Komodo dragon, and stevia. (G) The number of unique protein variants (i.e., nonsynonymous variants) using the dbSNP- and pseudoDB-based workflows on human, rice, sheep, chickpea, cattle, and African oil palm genomes. (H) Comparison of the number of unique variants called (in thousands, K) that are located in the coding region (red), 1 kb from the transcriptional start site (proximal, orange), and 10 kb from the transcriptional start site (distal, yellow).

Consistent with our results with rice and sheep, both AvgBQ and estimated error rates for cattle 1KBGP data improved using dbSNP- and pseudoDB-based recalibration (Figs 6A and S8C). For African oil palm, the trend is similar to our chickpea results, most likely because its dbSNP has not been updated since build 145. This improvement in cattle and African oil palm is corroborated by reduced technical bias after pseudoDB-based recalibration (S8D-S8E Fig) and substantial increase in additional genetic variants (Fig 6B). Notably, our pseudoDB workflow uncovered 563,288 additional variants in the African oil palm genome.

Brown bear, swan goose, Komodo dragon, and stevia are relatively under-studied species with limited resequencing data. Applying our pseudoDB workflow to brown bear, swan goose, and Komodo dragon, we found reduced technical bias (S8F Fig), improved AvgBQ (Fig 6C), and the identification of hundreds of thousands of additional variants (Fig 6D). With only two resequencing samples available, pseudoDB workflow found reduced technical bias (S8F Fig), improved AvgBQ (Fig 6E), and nearly 3 million additional genetic variants were discovered in the stevia genome (Fig 6F), altogether demonstrating that the pseudoDB workflow is applicable even to under-studied non-human species with limited genome resources.

Finally, we investigate the functional potential of these genetic variants by examining their genomic location with respect to protein-coding and promoter regions. Specifically, we focused on genetic variants that were uniquely identified with the standard dbSNP- or pseudoDB-based recalibration for human, rice, sheep, chickpea, cattle, and African oil palm. Recall that dbSNPs were unavailable for other non-human species. Of the newly identified variants, we focused on those in the coding region and nonsynonymous variants that result in protein variants (Fig 6G). Using the pseudoDB workflow, we discovered 10.7- to 42.8-fold more protein variants across these human and non-human genomes than when applying the standard dbSNP-based approach, including 35,354 chickpea protein variants. Beyond the coding region, we also uncovered the vast noncoding variants in the proximal promoters (i.e., 1 kb within transcriptional start site), and distal regions (i.e., 10 kb within transcriptional start site) (Fig 6H). With the same human resequencing data, the dbSNP- and pseudoDB-based approaches resulted in 39,798 and 99,056 unique noncoding variants, respectively. Similarly, our pseudoDB workflow identified a factor of 135.5-, 83.5-, 38.3-, 45.0-, and 22.5-fold more unique noncoding variants than dbSNP for rice, sheep, chickpea, cattle, and African oil palm, respectively. Altogether, this provides thousands of immediately testable hypotheses for understanding the genetic diversity in these non-human populations.

## 3. Discussion

The development of computational tools for recalibration and variant calling has led to the identification of 150 million human genetic variants and subsequently their functional role in phenotypic diversity and disease risk factors [54]. However, these success stories with human data have been relatively marginal with non-human data [28,30]. That is, despite the surge in non-human data generation and its data availability [21,42]. In this study, we hypothesized that this gap between human and non-human data might be computational and largely due to its limitation in applicability for non-human data. To assess this, we developed two performance metrics called Average Base Quality Score (AvgBQ) and Total Empirical Error Rate which traces the impact of computational recalibration on human and non-human data. With these metrics and other standard metrics, we find that computational recalibration overestimates the technical noise in non-human data which subsequently leads to suboptimal results for variant calling on non-human data.

To address this gap, we introduce a simple and portable solution for recalibration and variant calling on non-human data, including from rice (3KRGP) [1], sheep (ISGC) [40], and chickpea (ICRISAT) [3], cattle (1KBGP) [7], brown bear (NCBI:PRJNA1139383, n = 40) [47], swan goose (NCBI:PRJNA722049, n = 27) [48], African oil palm (ENA:PRJEB21246, n = 24) [49], Komodo dragon (NCBI:PRJNA738464, n = 24) [50], and stevia (NCBI:PRJNA684944, n = 2) [51]. Specifically, we focus on computational recalibration and provide detailed guidelines for the construction of a pseudo-database (pseudoDB) to circumvent the reliance on known variant databases in computational recalibration. Systematic evaluations show that the pseudoDB-based workflow is practical and convenient compared to relying on dbSNP or other known variant databases. Direct evaluation of variant calling accuracy with matched OvineSNP50 and sheep resequencing data demonstrate its applicability to a wide range of non-human genomes. Applying our pseudoDB-based workflow on human data from the Genome in a Bottle (GIAB) consortium [44,45], we find that computational recalibration is a necessary step for even high-depth and high-quality sequence data. More importantly, it also leads to statistical significant improvements when used with more advanced variant callers including DeepVariant [26], FreeBayes [24], and Strelka2 [25].

It is worth emphasizing that our workflow is fairly robust to the input data used for database construction. An important concern regarding the use of resequencing data for pseudoDB construction is the potential bias that might be inadvertently introduced during database construction. However, in practice, we find that this bias is marginal in terms of adjusting the AvgBQ and identifying genetic variants. The rice pseudoDB built with Indica rice data led to comparable results from when built on Japonica rice data. Data swapping analysis revealed that the use of an independent dataset resulted in only marginal improvement in performance compared to using the input data itself for pseudoDB construction. This is most likely due to the fact that the GATK3’s BaseRecalibrator assumes a relatively simple model of technical noise and tabulates sequencing data according to a list of features such as machine quality score and machine cycle [23]. In turn, this tabulation aggregates each source of technical error and marginalizes potential bias from the biological data of interest which fortunately helps avoid substantial overfitting from double-dipping.

Another important feature of our workflow is that it doesn’t rely on a particular genome version and provides a portable solution for alternative strains (e.g., Indica rice) [55–58], under-annotated genomes [59–61], and genome updates, such as for the recently gapless human genome [62,63]. Most computational tools have been developed for and evaluated with human data and on specific versions of the human reference genome [23,25,26,64,65]. On the contrary, the pseudoDB-based workflow does not rely on existing databases and compiles a list of genetic variants relative to the reference genome of interest. For example, to identify genetic variants of Indica rice, we can easily construct a pseudoDB based on the Indica rice genome (e.g., GCA_001623345.3). Thanks to long-read sequencing technology we now have more non-human genome sequences that are more complete [36,66–69]. We expect that the pseudoDB-based workflow will be applicable for these recent genome sequences for both human and non-human data, but the exact impact in terms of the number of contigs, the length of contigs, and genome coverage may require further investigation.

As for non-human data, we found the largest improvement in the number of genetic variants in chickpea (*Cicer arietinum*). Recent resequencing efforts of 3,171 cultivated chickpea accessions identified 3.94 million single-nucleotide polymorphisms (SNPs) [4]. Under our computational workflow, we identified 8.88 million SNPs with the standard dbSNP-based workflow and 11.18 million SNPs using pseudoDBs. This 25.9% increase in genetic variants with the same data highlights the importance of developing computational tools and systematic evaluations for non-human data. Of these, 35,232 SNPs correspond to nonsynonymous mutations that result in the translation of different protein variants. Further experimental investigation of these protein variants will help uncover the genetic basis of particular traits and features in non-human species [70–72]. Similar trends were consistently found in multiple under-studied non-human data with less than 20 sequencing data, thus supporting the general application of our pseudoDB workflow for non-human genomes [21]. Most variants that we identified lie within the non-coding genome, offering new avenues for systematic breeding and direct genetic engineering in the context of genome regulation [9,73].

## 4. Materials and methods

### 4.1. Resequencing data, reference genomes, and known variant databases

We collected genome resequencing data from 10 different species: human (*Homo sapiens*), rice (*Oryza sativa*), sheep (*Ovis aries*), chickpea (*Cicer arietinum*), cattle *(Bos taurus*), brown bear (*Ursus arctos*), swan goose (*Anser cygnoides*), African oil palm (*Elaeis guineensis*), Komodo dragon (*Varanus komodoensis*), and stevia (*Stevia rebaudiana*). Resequencing data were obtained from the 1000 Genomes Project (1KGP; https://www.internationalgenome.org/) [38,39,74], the 3000 rice genomes project (3KRGP; ENA:PRJEB6180) [1], the International Sheep Genomics Consortium (ISGC) (https://www.sheephapmap.org/; NCBI:PRJNA160933) [40], The International Crops Research Institute for the Semi-Arid Tropics (ICRISAT) (http://db.cngb.org/search/project/CNP0000370; NCBI:PRJNA362278) [3], and the 1000 Bull Genome Project (1KBGP) (NCBI:PRJNA238491) [7]. Other non-human species were collected from NCBI/ENA BioProject under accession numbers PRJNA1139383 (brown bear) [47], PRJNA722049 (swan goose) [48], PRJEB21246 (African oil palm) [49], PRJNA738464 (Komodo dragon) [50], and PRJNA684944 (stevia) [51]. All resequencing data were generated using Illumina short-read platforms albeit different generations. 1KGP human data were generated using the Illumina Genome Analyzer II. 3KRGP rice data, ISGC sheep data, ICRISAT chickpea data, 1KBGP cattle data, and African oil palm data were sequenced using the Illumina HiSeq 2000 platform. Brown bear and Komodo dragon were sequenced using the Illumina NovaSeq 6000 platform. Swan goose and stevia were sequenced using the Illumina HiSeq X Ten platform. The average genome coverage is approximately 5x for human, 9x for rice, 13x for sheep, 12x for chickpea, 11x for cattle, 16x for brown bear, 9x for swan goose, 1x for African oil palm, 18x for Komodo dragon, and 80x for stevia. The list of samples used in this analysis is available in S1 Table. Additional sheep resequencing data (PRJEB3138, n = 18, 13.1x genome coverage) with matched OvineSNP50 were obtained from Ensembl NextGen (ENA:PRJEB3138; Illumina HiSeq 2000 platform). The list of NextGen sheep samples used in this study is available in S2 Table.

The following genome references were used: the GRCh38 (*Homo sapiens*) obtained from the 1000 Genomes Project, the Nipponbare IRGSP-1.0 (GCA_001433935.1, *Oryza sativa*) obtained from the Rice Annotation Project (https://rapdb.dna.affrc.go.jp/) [75], the Oar_v4.0 (*Ovis aries*) obtained from NCBI (GCA_000298735.2), and the ASM33114v1 (*Cicer arietinum*) also obtained from NCBI (GCA_000331145.1), UMD_3.1.1 (*Bos taurus*) obtained from NCBI (GCF_000003055.6), GooseV1.0 (*Anser cygnoides*) obtained from NCBI (GCF_002166845.1), UrsArc2.0 (*Ursus arctos*) obtained from NCBI (GCF_023065955.2), EG5 (*Elaeis guineensis*) obtained from NCBI (GCF_000442705.1), ASM479886v1 (*Varanus komodoensis*) obtained from NCBI (GCF_004798865.1), ASM993640v2 (*Stevia rebaudiana*) obtained from NCBI (GCA_009936405.2).

We also used dbSNP which is a publicly accessible database of known genetic variants [31]. Initially constructed for human data, dbSNP has expanded to other species and has been irregularly updated in a series of builds. In detail, we used build 151 for human (GRCh38, n = 634,789,840), Japonica rice (IRGSP-1.0, n = 12,185,000), sheep (Oar_v4.0, n = 68,381,000), and build 146 for chickpea (ASM33114v1, n = 327,000), build 150 for cattle (UMD_3.1.1, n = 102,256,640), and build 145 for African oil palm (EG5, n = 52,383). It is worth mentioning that dbSNP stopped accepting non-human genetic variants as of Sept. 1, 2017.

### 4.2. Data preprocessing and variant calling for resequencing data

To discover genetic variants from resequencing data, we used a collection of computational tools available in the Genome Analysis Toolkit (GATK) version 3.8-1 [17]. Briefly, resequencing data were mapped to the corresponding reference genome using BWA MEM version 0.7.12 [76]. MarkDuplicates and SortSam in Picard version 1.118 were used to remove duplicates and sort read alignments (http://broadinstitute.github.io/picard/). Next, the raw (or machine-based) base quality scores were adjusted using GATK3’s BaseRecalibrator and PrintReads to account for technical errors made by the sequencing machine [23]. Of note, GATK3’s BaseRecalibrator builds a statistical model that handles various sources of technical variation such as read group, machine cycle, and nucleotide context. It is worth emphasizing that this recalibration step utilizes a statistical data binning approach and groups sequencing reads with similar technical features. Finally, the model-adjusted base quality scores were used to detect short genetic variants such as single-nucleotide (SNPs) and insertion/deletion polymorphisms (indels) using GATK3’s UnifiedGenotyper. The wall-clock runtime for a typical complete run starting from FASTQ files (e.g., 11.1 GB) is approximately 11.3 hours, including 2.5 hours for sequence alignment, 3.0 hours for pseudoDB construction, 3.1 hours for base quality score recalibration (BQSR), and 2.7 hours for variant calling. The recommended high-performance computing server for the pseudoDB workflow includes 128 GB RAM, 4 TB of disk space, 32 CPU threads at 2.4 GHz, and a recent stable Unix-based operating system.

As for other computational tools for variant calling, we utilized DeepVariant v1.8.0 [26], FreeBayes v1.3.8 [24], and Strelka2 v2.9.10 [25]. Specifically, variant calling was applied using DeepVariant for short-read whole genome sequencing data (--model_type = WGS), FreeBayes with computing variant qualities (--genotype-qualities), and Strelka2 with its default configuration script (i.e., configureStrelkaGermlineWorkflow.py). The wall-clock time via 32 CPU threads of 2.4 GHz on GIAB HG001 300x data were 9.5 hours, 3.3 hours, and 5.9 hours for DeepVariant, FreeBayes, and Strelka2, respectively. The max memory usage was on average 10.1 GB, 13.7 GB, and 0.7 GB for DeepVariant, FreeBayes, and Strelka2, respectively.

### 4.3. Gold standard for recalibration and variant calling on human data

High-depth human sequencing data and its respective genetic variants were obtained from the Genome in a Bottle (GIAB) consortium (https://www.nist.gov/programs-projects/genome-bottle) [44, 45]. Briefly, the GIAB sample HG001 and HG002 were sequenced on the Illumina HiSeq 2500 platform using 2x148nt paired-end reads and of a total coverage of 293.65x and 299.43, respect to GRCh38. Subsampling analyses of the HG001 and HG002 sequencing data were conducted at 30x coverages using SAMtools v1.21 [77] with a fixed random seed of 100 and corresponding subsampling rates (-s 100.1).

To assess the performance of recalibration and variant calling, we incorporated the HG001 and HG002 v4.2.1 benchmark from the GIAB consortium [45]. In detail, bcftools v1.21 [77] was used to extracted single-nucleotide variants (--types snps) that are either heterozygous or homozygous alternate (-i ‘GT = “alt"&(GT = “hom”|GT = “het”)’) from the HG002 benchmark and used as the gold standard for recalibration and variant calling on human data. Then, GATK3’s GenotypeConcordance v3.8-1 was applied to compare the genomic coordinates and genotypes of variants in confident regions between the gold standard and the computational results from different combinations of recalibration (raw, dbSNP, and pseudoDB) and variant calling using GATK3’s UnifiedGenotyper, DeepVariant, FreeBayes, and Strelka2.

### 4.4. The pseudo-database construction and approach for recalibration

Not accounting for technical sources of variation leads to a decrease in statistical power and in the context of variant discovery its ability to discover true genetic variants. A key prior information that Base Quality Score Recalibration (BQSR) utilizes for model-adjusted recalibration is the empirical quality score $Q_{Empir}$ [23]. The empirical quality score is estimated by comparing the mapped sequence read and the reference genome. Specifically, for each read group (or bin), the empirical quality score $Q_{Empir}$ is:


$$\begin{array}{c} Q_{Empir}\;=\;-\text{1}\text{0}log_{\text{1}\text{0}}(\hat{p}) \end{array}$$

$$\begin{array}{c} \hat{p}\;=\;(m+\text{1})/(n+\text{2}), \end{array}$$

where m corresponds to the number of mismatches and indels, and n corresponds to the number of nucleobases. Without accounting for genetic variation, this estimation assumes that all mismatches correspond to sequencing errors, which underestimate the true base quality score. Therefore, BQSR utilizes known variant databases (e.g., dbSNP) and excludes genetic loci that are known to vary in the population.

Here, we propose a computational approach for pseudo-database (pseudoDB) construction that circumvents the need for known variant databases in empirical quality score estimation and base quality score recalibration. PseudoDBs are generated solely from resequencing data through a sequence of optimization steps (S2A Fig). The pseudoDB is initialized by first calling genetic variants from unadjusted quality scores. Then, we re-run the variant calling pipeline with this initial pseudoDB. These incremental iterations may be run until base quality scores converge. In practice, we find that a single iteration is both necessary and sufficient for this purpose (S2C-S2F Fig). Codes for pseudoDB construction are available on Github (https://github.com/infoLab204/pseudoDB/). The pseudoDBs for human (GRCh38), rice (IRGSP-1.0, Japonica rice), sheep (NCBI:Oar_v4.0), chickpea (NCBI:ASM33114v1), cattle (NCBI:UMD_3.1.1), brown bear (NCBI:UrsArc2.0), swan goose (NCBI:GooseV1.0), African oil palm (NCBI:EG5), Komodo dragon (NCBI:ASM479886v1), and stevia (NCBI:ASM993640v2) are publicly available on Zenodo (https://doi.org/10.5281/zenodo.18463391) for further re-analysis of resequencing data and the discovery of genetic variants.

### 4.5. Systematic evaluation of computational recalibration

To quantitatively evaluate the efficacy of recalibration and variant calling, we defined a set of evaluation metrics for each computational step: (1) the Average Base Quality Score (AvgBQ), (2) the Total Empirical Error Rate. Details of the two metrics are described below.

#### 4.5.1. Average Base Quality Score (AvgBQ).

As with any high-throughput machine or device, modern sequencing machines are susceptible to unexpected systematic and technical errors. This is particularly critical for variant calling as it aims to identify true genetic variation from unwanted sequencing errors [12]. The quality score for each single nucleobase is subject to various sources of technical error and bias in terms of model-based recalibration. According to the law of large numbers in probability theory, AvgBQ approximates the expected base quality score that is intrinsic to the technological limits of the sequencing machine. In other words, AvgBQ must be independent of the organism or species being sequenced. To monitor the effect of statistical adjustment or recalibration of each nucleobase quality score, we compute the average (i.e., arithmetic mean) base quality score for each sample. Specifically, we compute the average Phred quality score $Q_{Phred}$ such that:


$$\begin{array}{c} Q=\frac{\text{1}}{n}\;\sum_{i}Q_{Phred} \end{array}$$

$$\begin{array}{c} Q_{Phred}\;=\;-\text{1}\text{0}\;{log}_{\text{1}\text{0}}p \end{array}$$

where p is the probability of observing an incorrectly called base reported by the sequencing machine [78], i is a nucleotide position of the reference genome, and n is the length of the reference genome.

#### 4.5.2. Total Empirical Error Rate.

We compute the total empirical error rate for each sample based on the variant database of interest. As mentioned above, it is essential that the empirical quality score $Q_{Empir}$ accounts for potential genetic variation in the resequencing data. Therefore, the completeness of the variant database determines the accuracy of estimating $Q_{Empir}$. To quantitatively evaluate variant databases, we compute the total empirical error rate, which is the number of potential genetic variants over nucleobase mismatches between the mapped reads and reference sequence. Specifically, for a given resequencing sample and database d, the total empirical error rate $\alpha_{d}$ is:


$$\begin{array}{c} \alpha_{d}=k_{d}\;/\;m, \end{array}$$

where $k_{d}$ represents the number of mismatches and indels that are not listed in database d, and m represents the number of mismatches. In other words, with a complete database of genetic variants, the total empirical error rate corresponds to the minimum error rate of the sequencing machine. Note that this metric is related to, but different from, $Q_{Empir}$. The total empirical error rate measures the type I error rate (or 1 - specificity), and the $Q_{Empir}$ is a proxy for the type II error rate (or 1 - sensitivity) of the computational pipeline.

### Financial disclosure

This work was supported by the Basic Science Research Program through the National Research Foundation (NRF) of Korea funded by the Ministry of Science and ICT (MSIT) [RS-2023–00261903 and RS-2024–00348305 to Y.-s.L.]; and funded by the Ministry of Education [RS-2025–25404480 to S.h.K and C.-y.L.]. This work was also supported by the Bio&Medical Technology Development Program of the National Research Foundation (NRF) funded by the Korean government (the Ministry of Science and ICT) [RS-2025–02216696 to Y.-s.L.]; and the Korea Bio Data Station (K-BDS) with computing resources including technical support [Y.-s.L.]. The funders had no role in study design, data collection and analysis, decision to publish, or preparation of the manuscript.

## Supporting information

S1 Fig Database dependent computational base quality score recalibration and number of variants in each dbSNP build. (A) Detailed workflow for modeling various sources of technical error. (B) Distribution of the model-adjusted average base quality score (AvgBQ) based on different builds of dbSNP database. Number of dbSNP variants of (C) human (*Homo sapiens*), (D) rice (*Oryza sativa*), (E) sheep (*Ovis aries*), and (F) chickpea (*Cicer arietinum*).
(PDF)

S2 Fig Optimization of the pseudo-database (pseudoDB) construction in terms of additional variants after computational recalibration and AvgBQ for human resequencing data. (A) The computational workflow for pseudoDB construction. Of note, no known variant databases were used for pseudoDB construction. (B) Number of additional variants (in millions, M) filtered by genotype quality (GQ) based on pseudoDBs that were constructed from different numbers of sequencing samples. The horizontal bar represents the number of additional variants after dbSNP-based recalibration. (C-F) The distributions of AvgBQ after additional iterations of pseudoDB construction for (C) 1KGP human data, (D) 3KRGP rice data, (E) ISGC sheep data, and (F) ICRISAT chickpea data, respectively. The X-axis indicates the number of iterations to construct each pseudoDB for recalibration. The horizontal bar represents the base quality score based on median AvgBQ using on their corresponding dbSNPs.
(PDF)

S3 Fig Technical noise in high-depth and high-quality human data. (A) Comparison of reported and empirical quality scores for 300x and 30x coverage of GIAB HG001 human data. Dashed diagonal lines indicate the identity function. RMSE: Root mean square error. (B) Comparison of reported and empirical quality scores of 1KGP human data for raw and recalibrated reads by pseudoDBs constructed by DeepVariant, Strelka2, and FreeBayes.
(PDF)

S4 Fig The importance of computational recalibration in rice, sheep, and chickpea. (A) Distribution of the adjusted AvgBQ with noDB-, dbSNP-, or pseudoDB-based recalibration for rice (3KRGP, n = 180), sheep (ISGC, n = 35), and chickpea (ICRISAT, n = 150). (B) Their total empirical error rate based on dbSNP and pseudoDB. The point represents the mean of the empirical error rate, and the error bars indicate its standard deviation. (C) Number of additional variants called (in thousands, K) after computational recalibration.
(PDF)

S5 Fig Benchmark performance of computational pipelines for recalibration and variant calling on the GIAB HG002 v4.2.1 benchmark. Recall with recalibration and variant calling applied to GIAB HG001 human data at different sequencing depths (30x, 100x, and 300x) using (A) GATK3's UnifiedGenotyper, (B) DeepVariant, (C) FreeBayes, and (D) Strelka2. Distribution of recall when applied to 1KGP human data (n = 150) using (E) GATK3's UnifiedGenotyper, (F) DeepVariant, (G) FreeBayes, and (H) Strelka2. White horizontal bars indicate its median precision. *P  <  0.05, **P  <  0.01, ***P  <  0.001, ****P  <  0.0001, n.s.: not significant; one-sided Wilcoxon-Mann-Whitney test.
(PDF)

S6 Fig Benchmark performance of computational workflows for recalibration and variant calling on the GIAB HG001 and HG002 v4.2.1 benchmark. Precision on GIAB HG002 v4.2.1 benchmark with recalibration and variant calling applied to different sequencing-depth (30x, 100x, and 300x) of GIAB HG002 human data using (A) GATK3's UnifiedGenotyper, (B) DeepVariant, (C) FreeBayes, and (D) Strelka2. Precision on GIAB HG001 v4.2.1 benchmark with recalibration and variant calling applied to different sequencing-depths (30x, 100x, and 300x) of GIAB HG001 human data using (E) GATK3's UnifiedGenotyper, (F) DeepVariant, (G) FreeBayes, and (H) Strelka2. Distribution of precision for 1KGP human data (n = 150) on GIAB HG001 v4.2.1 benchmark using (I) GATK3's UnifiedGenotyper, (J) DeepVariant, (K) FreeBayes, and (L) Strelka2. White horizontal bars indicate its median precision. *P  <  0.05, **P  <  0.01, ***P  <  0.001, ****P  <  0.0001, n.s.: not significant; one-sided Wilcoxon-Mann-Whitney test.
(PDF)

S7 Fig Systematic evaluation of pseudoDB workflow and data swapping analysis for pseudoDB construction. (A) Total empirical error rate based on dbSNP and pseudoDB for sheep resequencing data (PRJEB3138, n = 18) from the Ensembl NextGen project. The point represents the mean of the empirical error rate, and the error bars indicate its standard deviation. (B) Comparison of reported and empirical quality scores of sheep resequencing data. Dashed diagonal lines indicate the identity function. RMSE: Root mean square error. (C) Schematic of the data swapping analysis to quantify the effect of double-usage of input data for pseudoDB construction. HG001 and H002 resequencing data of GIAB was used. HG001 v4.2.1 variants from the GIAB consortium were used as a benchmark. dbSNP: Standard recalibration of HG001 resequencing data using the external dbSNP database. pseudoDB(Non-self): Recalibration of HG001 resequencing data using a pseudoDB database constructed from an independent data HG002. pseudoDB(Self): Recalibration of HG001 resequencing data using a pseudoDB database constructed from HG001 data itself. neg.ctrl: Recalibration of the independent data HG002 using a pseudoDB constructed from the standard pseudoDB approach which is the HG002 data. (D) The precision and recall of genetic variants called by the dbSNP, pseudoDB(Non-self), pseudoDB(Self), and neg.ctrl pipelines. HG001 v4.2.1 variants from the GIAB consortium were used as the gold standard. GQ: Genotype Quality.
(PDF)

S8 Fig Systematic evaluation of pseudoDB workflow on additional non-human data. Number of dbSNP variants of (A) cattle (*Bos taurus*) and (B) African oil palm (*Elaeis guineensis*). (C) The total empirical error rate based on dbSNP and pseudoDBs in 1KBGP cattle data (n = 146) and African oil palm data (PRJEB21246, n = 12). The point represents the mean of total empirical error rate, and the error bars indicate its standard deviation. Comparison of reported and empirical quality scores of (D) 1KBGP cattle data, (E) African oil palm data, and (F) brown bear data (PRJNA1139383, n = 20), swan goose data (PRJNA722049, n = 14), Komodo dragon data (PRJNA738464, n = 12), and stevia data (PRJNA684944, n = 1). Dashed diagonal lines indicate the identity function. RMSE: Root mean square error.
(PDF)

S1 Table Human and non-human resequencing data used in this study. (XLSX)

S2 Table Sheep SNP array and resequencing data from the Ensembl NextGen project used in this study. (XLSX)
