# Supplementary material for: A portable recalibration workflow for reference-based variant calling in non-human genomes
Source: PLoS Comput Biol. 2026 Aug 20;22(8):e1014603. doi: 10.1371/journal.pcbi.1014603 (PMC13492802; doi:10.1371/journal.pcbi.1014603)

Supplementary Figure 8

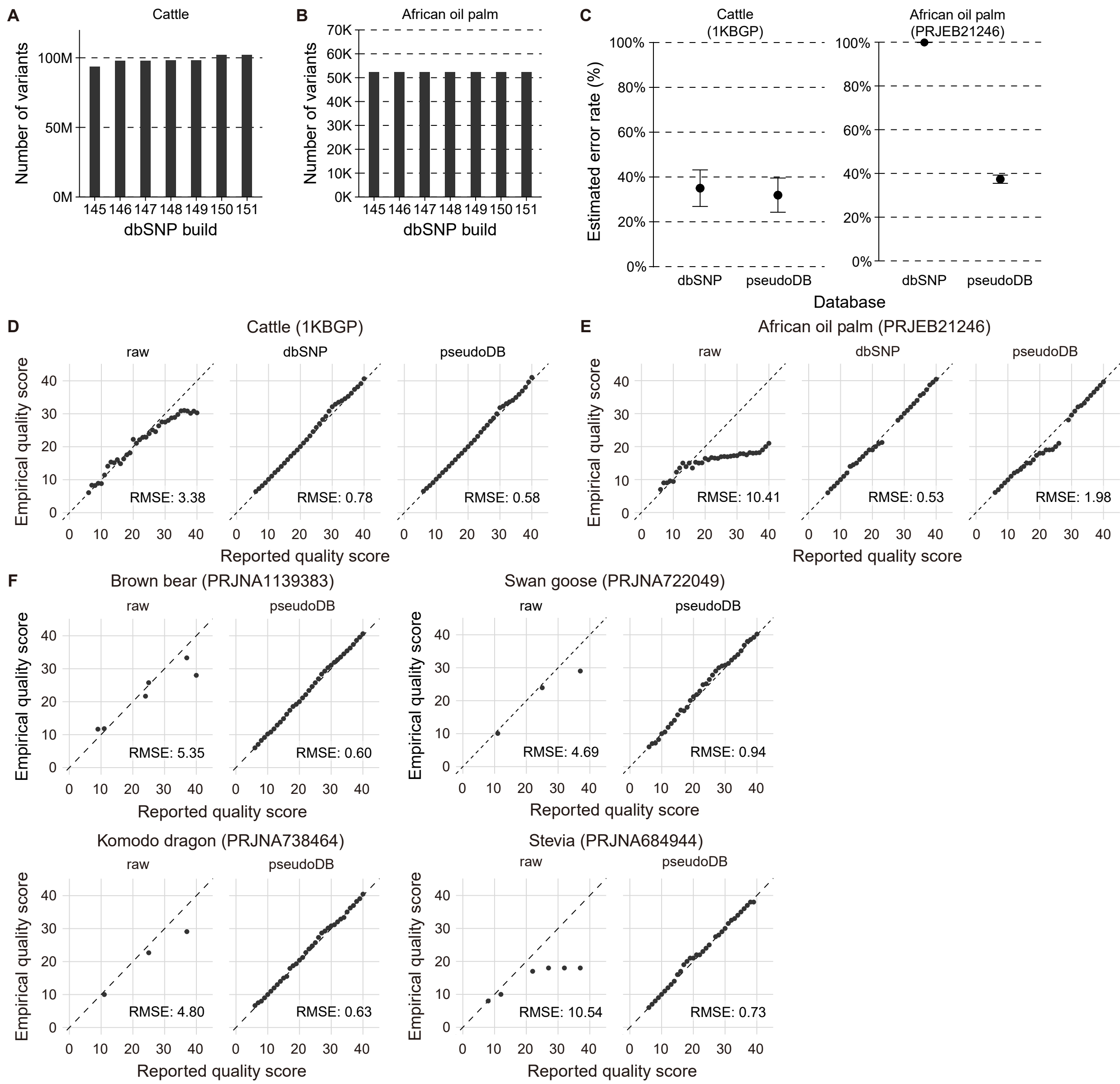

Supplement: Supplementary file 8 [file pcbi.1014603.s008.pdf]
